# Comparative biochemical responses of sorghum (*Sorghum Bicolor* L. Moench) and rice (*Oryza Sativa* L.) to simulated caterpillar herbivory: alterations in soluble sugars and phenolic metabolism associated with induced defense

**DOI:** 10.1080/15592324.2026.2758546

**Published:** 2026-11-03

**Authors:** Cyprian Osinde, Clement Nyakoojo, Agnes Nandutu Masawi, Hannington Oryem-Origa, Arthur K. Tugume, Anthony M. Nsubuga, Jamilu Edrisa Ssenku

**Affiliations:** a Faculty of Agriculture and Environmental Sciences, Muni University, Arua, Uganda; b Department of Plant Sciences, Microbiology and Biotechnology, College of Natural Sciences, Makerere University, Kampala, Uganda; c Department of Biology, Mountains of the Moon University, Fort Portal, Uganda; d Department of Biochemistry and Systems Biology, College of Natural Sciences, Makerere University, Kampala, Uganda

**Keywords:** Simulated herbivory, *Mythimna loreyi*, Epuripur Sorghum NERICA 9 rice phenolic acids, soluble sugars, induced defense plant metabolism

## Abstract

Herbivore-associated stimulation can alter primary and specialized metabolism, but comparative biochemical responses of cereal crops remain insufficiently characterized. This study investigated changes in soluble sugars and targeted phenolic acids in sorghum (*Sorghum bicolor* L. Moench) cultivar Epuripur sorghum and NERICA 9 rice following simulated caterpillar herbivory. Plants were subjected to mechanical leaf wounding followed by application of diluted oral secretions from fourth- and fifth-instar *Mythimna loreyi* larvae (WOS), while untreated plants served as controls. After 24 h, leaf tissues were analyzed by high-performance liquid chromatography (HPLC) for glucose, fructose, and seven targeted phenolic acids. Two-way ANOVA evaluated effects of plant material, treatment, and their interaction. WOS treatment significantly reduced glucose and fructose concentrations in both materials (*P* < 0.001), with significant plant material × treatment interactions. Several phenolic acids increased or became detectable following WOS treatment, including *p*-hydroxybenzoic, vanillic, syringic, *p*-coumaric, cinnamic, and chlorobenzoic acids. Significant plant material × treatment interactions occurred for most phenolic acids, whereas the interaction for *p*-coumaric acid was not significant (*P* = 0.205). Epuripur sorghum generally exhibited higher concentrations of several metabolites than NERICA 9. These findings indicate material-specific biochemical responses to simulated caterpillar-associated stimulation involving changes in both primary and phenolic metabolism. However, the study does not establish carbon allocation, growth–defense trade-offs, or direct insect resistance. Because wounding-only and oral-secretion-only treatments were not included, their individual effects could not be separated. The results provide candidate biochemical responses for further investigation using direct herbivore feeding, multiple genotypes, time-course sampling, and physiological and insect-performance measurements.

## Introduction

### Importance of Sorghum and rice and herbivore pressure

Sorghum (*Sorghum bicolor* L. Moench) and rice (*Oryza sativa* L.) are globally important cereal crops that contribute substantially to food security, livestock production, and agricultural livelihoods. Sorghum is particularly important in semi-arid and tropical production systems, whereas rice is a major staple crop across Asia and Africa.^1^ ^,^ ^2^

Both crops are attacked by numerous insect herbivores, including lepidopteran larvae. Stem-boring insects are economically important cereal pests because larval feeding within plant tissues can disrupt vascular transport, weaken plants, and reduce productivity.^3^ ^,^ ^4^ The present experiment, however, did not involve direct feeding by a stem-borer. Instead, it used mechanical leaf injury combined with oral secretions from the rice armyworm, *Mythimna loreyi*. Accordingly, the study is framed as an investigation of general simulated caterpillar-herbivory responses rather than a direct assessment of stem-borer resistance.

*Mythimna loreyi* is a polyphagous noctuid herbivore associated with gramineous crops, including rice and other cereals.^5^ Its use in the present experiment provides an elicitor-based system for examining plant biochemical responses to caterpillar-associated stimulation, but it does not reproduce the feeding behavior, feeding location, or physiological consequences of a stem-boring insect.

Understanding biochemical responses to herbivory remains important because induced defense mechanisms can contribute to plant resistance and tolerance and may ultimately provide traits useful for integrated pest management and crop improvement.^6^ ^,^ ^7^

#### Plant-induced responses to caterpillar herbivory

Caterpillar-associated injury can trigger a coordinated suite of local and systemic responses involving wound signaling, phytohormonal regulation, redox changes, and reorganization of primary and specialized metabolism. Caterpillar feeding causes mechanical tissue damage and introduces herbivore-associated molecular signals that can activate calcium fluxes, reactive oxygen species (ROS), mitogen-activated protein kinase cascades, and phytohormonal pathways involving jasmonates, salicylic acid, and ethylene.^8‐10^

Oral secretions from herbivores can contain elicitors that modify plant responses to mechanical damage. Compounds present in caterpillar oral secretions can enhance herbivore-induced volatile production and downstream defense signaling.^11^ ^,^ ^12^ Studies in rice have also shown that simultaneous perception of different herbivore-associated elicitors can influence defense responses.^13^ Activation of these signaling pathways can result in extensive changes in both primary and secondary metabolism, reflecting metabolic demands associated with growth, repair, signaling, and defense.^14‐16^ Recent work in sorghum has further demonstrated that simulated herbivory can alter herbivore-induced volatile emissions and activate volatile-mediated interactions with natural enemies. In particular, Osinde et al.^17^ showed that mechanical wounding combined with *Chilo partellus* oral secretions increased the emission of candidate herbivore-induced plant volatiles, including DMNT and (Z)-3-hexenyl acetate, and that these compounds were behaviorally active toward the parasitoid *Cotesia flavipes*. These findings highlight that herbivore-associated stimulation can affect not only local biochemical metabolism but also volatile-mediated indirect defense signaling.

In the present experiment, mechanical wounding and oral secretion application were combined to generate a simulated caterpillar-herbivory treatment. Because wounding-only and oral-secretion-only treatments were not included, the relative contributions of physical injury and oral-secretory components cannot be resolved. Furthermore, metabolites were measured at a single time point, and growth and carbon flux were not assessed. Therefore, metabolite changes are interpreted as biochemical responses consistent with metabolic reprogramming rather than direct evidence of a growth–defense trade-off.

### Phenolic compounds and plant defense

Phenolic compounds comprise diverse secondary metabolites, including phenolic acids, flavonoids, and tannins, which contribute to plant defense and responses to herbivory.

Phenolic metabolites may influence herbivore interactions through effects on digestibility, digestive enzymes, oxidative balance, tissue structure, and other physiological processes.^18‐21^

Phenolic acids such as *p*-hydroxybenzoic acid, vanillic acid, syringic acid, *p*-coumaric acid, and cinnamic acid occur in plant tissues and may change following biotic stress. Several are associated with phenylpropanoid metabolism and can contribute to cell-wall-associated processes and antioxidant responses.^15^ ^,^ ^22^ ^,^ ^23^

### Soluble sugars and defense-associated metabolism

Soluble carbohydrates are important components of plant metabolism because they provide energy and carbon skeletons required for growth and biosynthesis. They can also function as signaling molecules that influence defense responses.^16^ ^,^ ^24^ Herbivore-associated stress can therefore modify primary metabolism, resulting in changes in sugar pools and other metabolites.

A decrease in soluble sugar concentration following herbivore-associated stimulation may be consistent with altered carbohydrate utilization, but a decrease alone does not demonstrate carbon reallocation toward defense. Direct evidence of carbon allocation would require measurements such as carbon flux, total carbon pools, photosynthetic assimilation, respiration, or isotopic tracing. The concept of growth–defense trade-offs involves allocation and regulatory relationships between growth and defense processes and cannot be inferred from a change in a single metabolite pool alone.^25^

### Comparative Physiological context of Sorghum and rice

Sorghum uses C₄ photosynthesis whereas rice uses C₃ photosynthesis. C₄ plants generally have physiological advantages under conditions of high temperature and irradiance because of reduced photorespiration and enhanced carbon fixation efficiency.^26^ ^,^ ^27^ These differences may influence primary metabolism and resource availability.

Sorghum contains diverse phenolic metabolites, flavonoids, tannins, and other specialized metabolites associated with responses to environmental and biotic stress.^22^ ^,^ ^28^ Rice also possesses extensive inducible defense mechanisms, although the magnitude and composition of metabolic responses may vary among genotypes and environmental conditions. Differences between one sorghum genotype and one rice material therefore cannot be generalized to the two crop species as a whole.

Previous work has demonstrated differences in herbivore-induced volatile emissions between sorghum and rice.^29^ Subsequent work in sorghum demonstrated that selected herbivore-induced volatiles, particularly DMNT and (Z)-3-hexenyl acetate, were behaviorally active toward the parasitoid *Cotesia flavipes*, providing evidence that simulated herbivory can influence both volatile emission and indirect defense signaling.^17^ However, comparative biochemical responses involving soluble sugars and individual phenolic acids following artificial caterpillar herbivory have received less attention in these two cereal materials.

### Knowledge gap and study objectives

Understanding biochemical responses associated with herbivore-associated stimulation may help identify candidate traits for future studies of crop resistance. While volatile-mediated responses have received considerable attention,^17^ ^,^ ^29^ ^,^ ^30^ changes in soluble sugars and phenolic-acid profiles provide complementary information on primary and specialized metabolism. Our recent work demonstrated that sorghum subjected to simulated herbivory emitted candidate HIPVs, including DMNT and (Z)-3-hexenyl acetate, that elicited behavioral responses in the parasitoid *Cotesia flavipes.* ^17^ The present study extends this line of investigation by examining biochemical responses in primary and phenolic metabolism rather than volatile-mediated tritrophic interactions.

This study investigated the biochemical responses of sorghum cultivar Epuripur and rice material described as NERICA cultivar 9, a NERICA rice material developed for African production environments,^31^ following simulated caterpillar herbivory involving mechanical leaf wounding and *M. loreyi* oral secretions.

Specifically, we investigated:

1. Changes in soluble sugar concentrations following artificial herbivory.
2. Alterations in individual phenolic-acid profiles following treatment.
3. Differences in constitutive and treatment-associated biochemical profiles between the tested sorghum and rice materials.

We hypothesized that artificial herbivory would alter soluble sugar concentrations and increase phenolic-acid accumulation through stimulation of defense-associated metabolism. We further expected the magnitude of these biochemical changes to differ between the tested sorghum and rice materials, without assuming that such differences represent species-wide differences in resistance. The present study therefore does not constitute a direct test of stem-borer resistance. Rather, it evaluates biochemical responses of two cereal materials to a controlled simulated caterpillar-herbivory treatment consisting of mechanical injury and application of *M. loreyi* oral secretions.

## Materials and methods

### Plant materials and growth conditions

Seeds of sorghum (*Sorghum bicolor* L. Moench) cultivar Epuripur and rice (*Oryza sativa* L.) cultivar NERICA 9 (New Rice for Africa 9) were used. NERICA 9 is a rainfed upland rice cultivar developed through interspecific hybridization between Asian rice (*Oryza sativa* L.) and African rice (*Oryza glaberrima* Steud.) at the Africa Rice Center (AfricaRice).^32^ It was selected as the rice material because it belongs to the NERICA group developed for African production environments.^31^

Sorghum seeds were surface-sterilized and germinated on moist sterile filter paper for 7 days before uniform seedlings were transplanted into 100-cm^3^ plastic pots containing sterilized loam soil. Rice seeds were germinated directly in nutrient-rich loam soil, and 2-week-old seedlings were transplanted individually into similar pots containing sterilized field soil.

Plants were maintained in a greenhouse under a 12-h light/12-h dark photoperiod, at 28 ± 3 °C and approximately 80–120 μmol m^−2^ s^−1^ photosynthetically active radiation. Sorghum plants were irrigated every 2 days, whereas rice plants were maintained under flooded conditions in shallow water trays. Plants aged 4–6 weeks with comparable growth characteristics were selected for the experiments.

### Insect material and collection of oral secretions

Artificial herbivory was simulated using oral secretions obtained from fourth- and fifth-instar larvae of the rice armyworm, *Mythimna loreyi* Duponchel (Lepidoptera: Noctuidae). Larvae were maintained individually under controlled laboratory conditions and reared on fresh sorghum or rice leaves until the required developmental stage for oral-secretion collection. Five young cereal leaves were provided per rearing plate and replaced every two days to maintain fresh food material and adequate feeding substrate. No grain or formulated feed was used for larval rearing.

Individual larvae were gently restrained, and regurgitated oral secretions were collected using sterile micropipettes. Oral secretions collected from individual larvae were pooled to obtain a homogeneous oral-secretion preparation. The pooled preparation was gently mixed, aliquoted to minimize repeated freeze–thaw cycles, and stored at −80 °C until use. Aliquots were thawed on ice immediately before treatment and diluted three-fold with sterile deionized water. Because larvae were reared on fresh cereal leaves before oral-secretory collection, the collected oral secretions may have contained a mixture of insect-derived and diet-associated constituents. The present experiment was therefore designed to evaluate the response to the collected oral-secretory preparation rather than to attribute the observed effects to a specific purified insect elicitor.

### Simulated herbivory treatment

Artificial herbivory was simulated by mechanical wounding followed by application of *M. loreyi* oral secretions. The treatment was designated wounded plus oral secretion (WOS). The experimental treatment used in this study is simulated caterpillar herbivory rather than a direct measure of stem-borer resistance. The treatment combined mechanical leaf wounding with application of diluted oral secretions from the rice armyworm, *M. loreyi*, and did not involve direct feeding by stem-borer larvae. Accordingly, the biochemical responses reported here are interpreted as responses of the tested plant materials to this simulated caterpillar-herbivory treatment.

The two youngest fully expanded leaves of each plant were mechanically wounded along the midrib using a fabric pattern wheel. Immediately after wounding, 15 μL of three-fold diluted oral secretion was applied directly to the wounded areas. The treatment was repeated after two hours on the same damaged regions to simulate repeated herbivore-associated stimulation.

Control plants received neither mechanical wounding nor oral secretion. Plants were maintained for 24 h following treatment before tissue collection.

The experiment therefore comprised two experimental conditions: untreated control and WOS, with three independent biological replicates per treatment within each crop material. Because wounding-only and oral-secretion-only treatments were not included, the design does not permit separation of mechanical injury effects from oral-secretory effects or their interaction.

### Plant tissue collection

Leaf tissues were collected 24 h after treatment at approximately 17:00 h to minimize variation associated with diurnal metabolic changes. Leaves were excised at the petiole, weighed, immediately frozen in liquid nitrogen, transferred to sterile cryogenic tubes, and stored at −80 °C until extraction. Liquid nitrogen (≥99.999% purity; Oxygas Limited, Kampala, Uganda) was used for rapid tissue freezing and subsequent storage of samples prior to biochemical analysis. Rapid freezing and low-temperature storage were used to minimize post-harvest metabolic changes.

### Extraction and analysis of soluble sugars

Approximately 2.5 g of frozen leaf tissue was ground into a fine powder in liquid nitrogen and extracted with 50 mL of 80% methanol (v/v). Samples were homogenized and centrifuged at 12,500 × *g* for 20 min at 8 °C. Supernatants were filtered through 0.22-μm membrane filters before HPLC analysis. The concentrations obtained from the HPLC analysis were converted from extract concentration to tissue-normalized concentration using the measured extract volume, any dilution factor applied to the sample, and the fresh mass of tissue extracted. Final soluble-sugar concentrations are therefore expressed as μg g^−1^ fresh weight (FW).

The soluble sugars specifically quantified were glucose and fructose. Glucose and fructose were quantified using external calibration curves prepared from authentic reference standards. Calibration standards covered the concentration range used for the analytical measurements of the diluted extracts. Sample extracts were diluted where necessary so that the measured analyte concentrations fell within the validated calibration range. The concentration obtained from the calibration curve was converted to μg g^−1^ fresh weight (FW) using the dilution factor, total extraction volume, and mass of fresh tissue extracted. Final concentrations were recalculated on a fresh-weight basis as μg g^−1^ FW using the concentration measured in the extract, extraction volume, dilution factor, and mass of fresh tissue extracted. The original notation of μg L^−1^ FW was therefore corrected because μg L^−1^ describes the extract concentration rather than the tissue-normalized concentration.

### Extraction of phenolic compounds

Phenolic compounds were extracted using a procedure adapted from Castellari et al.^33^, with modifications for the targeted analysis of selected phenolic acids. Approximately 2.5 g of fresh leaf tissue was extracted with 50 mL of diethyl ether under continuous agitation for 24 h at room temperature. The extracts were filtered and centrifuged at 12,500 × *g* for 20 min at 8 °C. The organic phase was then collected, evaporated to dryness, and reconstituted in HPLC-grade methanol. The reconstituted extracts were filtered through 0.20-μm membrane filters before HPLC analysis.

Diethyl ether was retained from the Castellari et al.^33^-based procedure as the extraction solvent because it facilitates partitioning of non-polar matrix constituents, including neutral lipids, waxes, and other hydrophobic compounds, thereby reducing potential matrix interference during HPLC analysis. Its use was therefore intended as part of the method-derived extraction and matrix-cleanup strategy rather than as a response to high lipid content in the plant material. The procedure was designed for targeted determination of selected phenolic acids and was not intended to provide comprehensive recovery of all phenolic classes. Because extraction efficiency was not independently validated for individual phenolic acids or across different phenolic classes, the resulting data are reported as a targeted phenolic-acid profile rather than as total phenolic content or a comprehensive phenolic profile.

Phenolic acids were identified by comparing the retention times of sample peaks with those of authentic reference standards analyzed under identical chromatographic conditions. The retention times presented in Table 1 and Figure 1 were experimentally determined from the reference standards analyzed in the present study while Table 3 and Figure 2 represents values of phenolic acid concentrations and chromatogram peaks and retention time of phenolic compounds obtained from experimental samples.

**Table 1.** Calibration characteristics of glucose and fructose and retention times of targeted phenolic acids under the HPLC conditions used in this study.

Analyte	Calibration range (μg L^−1^)	R^2^	LOD (μg L^−1^)	RT (Retention time) (min)
Glucose	0.5–20	0.999	0.01	12.5
Fructose	0.5–20	0.999	0.03	13.4
*p*-Hydroxybenzoic acid	NA	NA	NA	19.403
Vanillic acid	NA	NA	NA	31.520
Caffeic acid	NA	NA	NA	32.491
Syringic acid	NA	NA	NA	33.899
*p*-Coumaric acid	NA	NA	NA	35.147
Cinnamic acid	NA	NA	NA	43.243
Chlorobenzoic acid	NA	NA	NA	46.496

Note: LOD, limit of detection; R^2^, coefficient of determination; NA, not assessed. Calibration range, R^2^, and LOD values are reported only for glucose and fructose because corresponding analytical-performance experiments were performed only for these analytes. Retention times for phenolic acids were obtained using authentic reference standards under the specified chromatographic conditions and are presented as identification characteristics rather than as evidence of complete analytical-method validation. Formal phenolic-acid LOD, LOQ, precision, accuracy/recovery, and robustness were not determined. The limits of detection (LOD) for glucose and fructose were estimated from the calibration data using the standard deviation of the analytical response and the slope of the corresponding calibration curve. The LOD was calculated according to the relationship LOD = 3.3 σ/S,^34^ where *σ* represents the standard deviation of the response and *S* represents the slope of the calibration curve. The resulting estimated LODs were 0.01 μg L^−1^ for glucose and 0.03 μg L^−1^ for fructose

**Figure 1.**
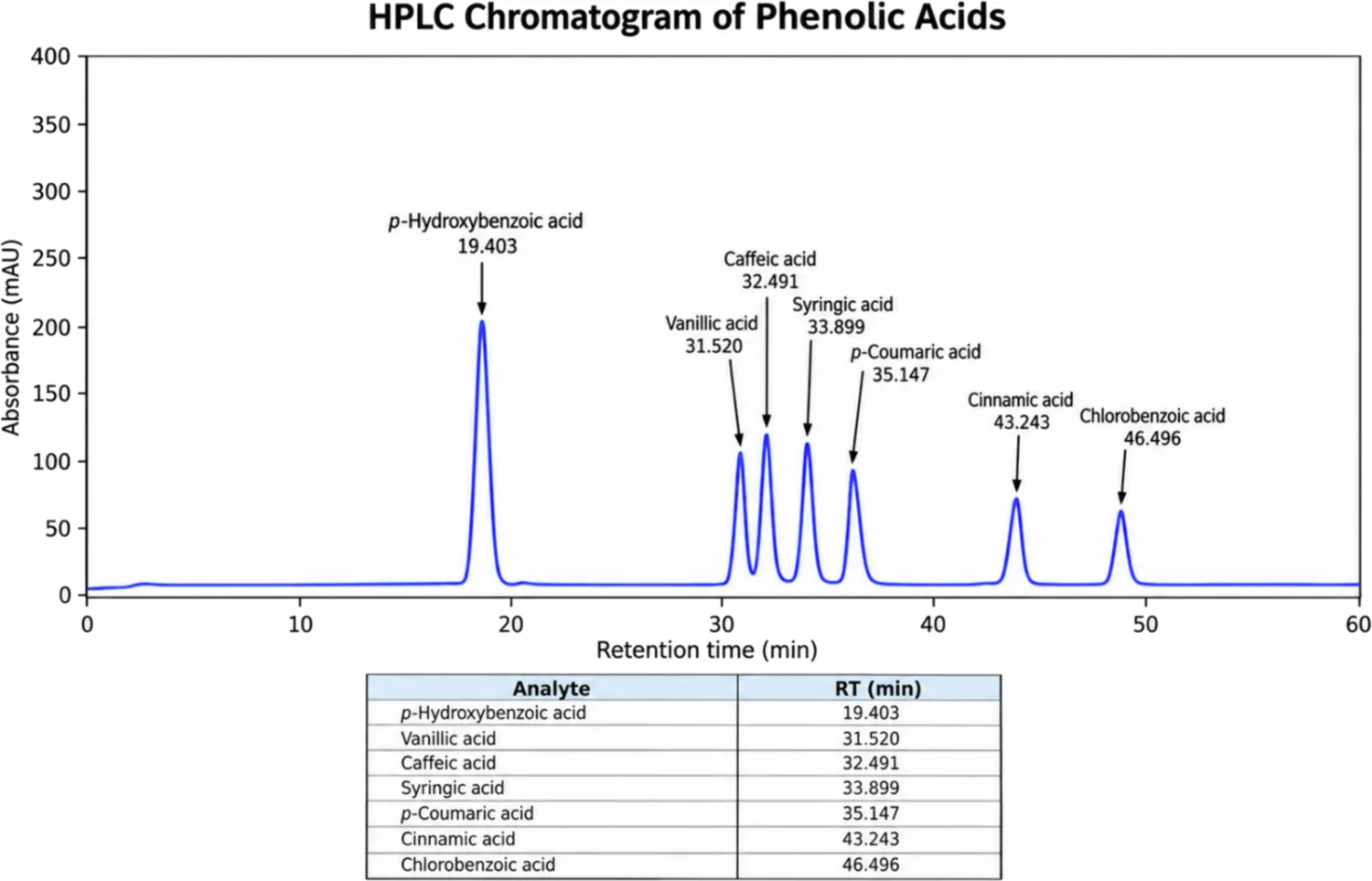
Chromatograms of standard phenolic Compounds used in the experiment.

**Figure 2.**
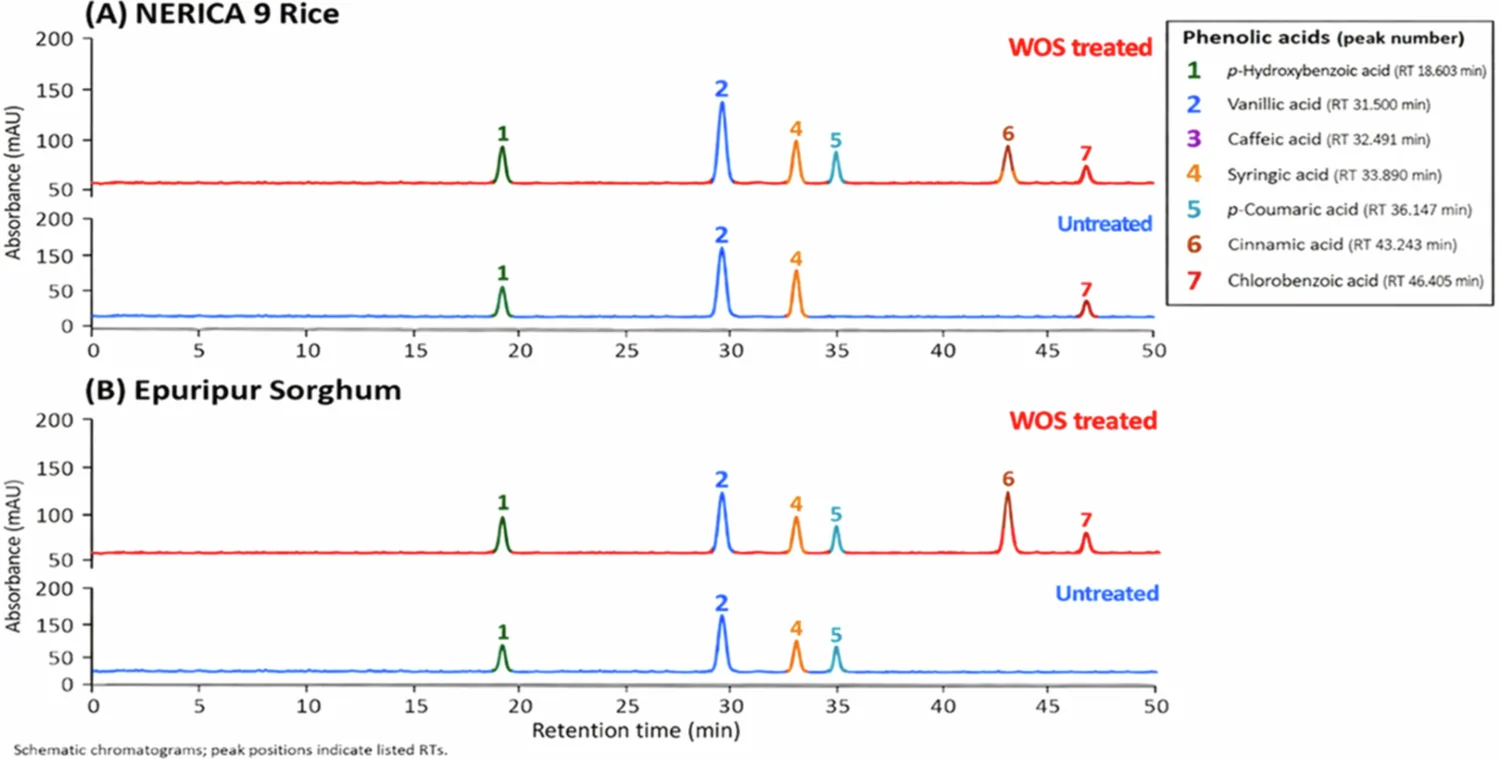
Chromatograms of different Compounds detected in Nerica 9 Rice, and Epuripur Sorghum.

### Phenolic standards

Analytical standards used for identification and quantification included *p*-hydroxybenzoic acid, vanillic acid, caffeic acid, syringic acid, *p*-coumaric acid, cinnamic acid, and chlorobenzoic acid. Standards had analytical purity greater than 98% and were prepared as serial dilutions.

The analytical method was designed to prioritize detection and quantification of targeted phenolic acids rather than characterization of the complete spectrum of phenolic metabolites. Accordingly, non-detection of a compound refers to the analytical conditions and detection capability of the method and should not be interpreted as proof that the compound is completely absent from the plant tissue.

Analytical reference standards used for identification of the targeted phenolic acids were *p*-hydroxybenzoic acid, vanillic acid, caffeic acid, syringic acid, *p*-coumaric acid, cinnamic acid, and chlorobenzoic acid. The standards were obtained from Sigma-Aldrich (Merck KGaA, Darmstadt, Germany) through Kobian Scientific (Uganda) Ltd., Kampala, Uganda, and were of analytical grade or high analytical purity (≥98%). Individual stock solutions were prepared in HPLC-grade methanol at known concentrations, and working solutions were prepared by serial dilution before HPLC analysis. The standards were analyzed under the same chromatographic conditions as the plant extracts, and peak identities were assigned primarily by comparison of retention times.

### HPLC analysis

Metabolite analysis was performed using a Jasco HPLC system (Tokyo, Japan) equipped with a PU-980 pump, UV-970 UV detector, and RI-830 refractive-index detector. Samples (20 μL) were injected using a Rheodyne 7125 injector and separated on an Aminex HPX-87H column (300 × 7.8 mm) fitted with a compatible guard column. Soluble sugars and phenolic acids were analyzed under separate chromatographic conditions.

For soluble-sugar analysis, the mobile phase consisted of 0.045 N sulfuric acid containing 6% acetonitrile and was delivered at a flow rate of 0.5 mL min^−1^. The column temperature was maintained at 55 °C, and detection was performed using the refractive-index detector. Glucose and fructose were the target soluble sugars. Calibration curves were prepared using standard solutions ranging from 0.5–20 μg L^−1^ for each analyte. The resulting concentrations were converted to tissue-normalized values and expressed as μg g^−1^ fresh weight (FW), accounting for the extract volume, dilution factor, and fresh tissue mass.

For phenolic-acid analysis, solvent A consisted of water/phosphoric acid (99.5:0.5, v/v), whereas solvent B consisted of acetonitrile/water/phosphoric acid (50:49.5:0.5, v/v/v). Separation was performed at a flow rate of 1.0 mL min^−1^ with the column maintained at 25 °C. Detection was carried out at 280 nm using the UV detector. The gradient program is provided in Table 2. Following each analysis, the column was re-equilibrated with the initial mobile-phase composition before the subsequent injection.

**Table 2.** Gradient elution program used for HPLC separation of phenolic acids.

Time (min)	Solvent A (%)	Solvent B (%)
0	100	0
2	100	0
7	80	20
25	60	40
31	60	40
35	20	80
40	0	100
42	0	100
47	100	0
62	100	0

Note: Solvent A, water/phosphoric acid (99.5:0.5, v/v); solvent B, acetonitrile/water/phosphoric acid (50:49.5:0.5, v/v/v). The flow rate was 1.0 mL min^−1^, column temperature was 25 °C, detection was at 280 nm, and the injection volume was 20 μL.

Peak identification was based primarily on comparison of sample retention times with those of authentic reference standards analyzed under the same chromatographic conditions. No co-injection experiments were performed; therefore, peak identity was based on retention-time comparison with authentic standards analyzed under the same chromatographic conditions. The experimentally determined retention times of the targeted phenolic acids are reported in Table 1. Phenolic compounds were detected at 280 nm using UV detection, whereas glucose and fructose were quantified using refractive-index detection. Phenolic-acid concentrations were estimated by comparison of sample peak responses with those of the corresponding authentic reference standards analyzed under the same chromatographic conditions. The chromatographic workflow was adapted from previously described HPLC approaches for carbohydrates and organic acids, including the method of Castellari et al.^33^, with modifications to accommodate the plant extracts and analytes examined in the present study. Because the present chromatographic conditions and sample matrix differed from those of the original method, the cited procedure is treated as a methodological basis rather than as validation of the present analytical method.

### Analytical performance assessment

Analytical performance assessment was limited to the parameters experimentally determined in the present study. Calibration curves for glucose and fructose were evaluated over 0.5–20 μg L^−1^ and showed strong linearity, with (R^2^ = 0.999) for both analytes. Limits of detection (LODs) were estimated from the calibration response using the relationship (LOD = 3.3σ/S), where *σ* is the standard deviation of the response and S is the slope of the corresponding calibration curve. The estimated LODs were 0.01 μg L^−1^ for glucose and 0.03 μg L^−1^ for fructose.

No experimentally established LOD or limit of quantification (LOQ) values were available for the individual phenolic acids. These compounds were identified using authentic reference standards and quantified under the specified chromatographic conditions; however, compound-specific validation of LOD, LOQ, precision, accuracy/recovery, specificity, and robustness was not performed. Consequently, the phenolic-acid measurements are considered targeted comparative analytical measurements rather than results from a comprehensively validated quantitative method.

A complete analytical validation according to ICH Q2(R2) was therefore not undertaken (International Council for Harmonization.^34^ In particular, the calibration and estimated LOD data obtained for glucose and fructose should not be extrapolated to the phenolic-acid measurements. Future studies should establish analyte- and matrix-specific LOD and LOQ values and evaluate precision, accuracy/recovery, specificity, robustness, and other relevant analytical performance characteristics for the phenolic acids.

### Statistical analysis

The experimental unit was an individually treated plant. Three independent plants were used for each plant material × treatment combination, resulting in four experimental groups with *n* = 3 biological replicates per group. Where replicate HPLC injections were performed, individual injections were treated as technical measurements and averaged before statistical analysis; thus, technical injections were not considered independent biological replicates.

Data were analyzed using two-way analysis of variance (ANOVA), with plant material (NERICA 9 rice and Epuripur sorghum) and treatment (untreated control and wounded plus oral secretion [WOS]) specified as fixed factors, together with their interaction. For analytes detected across the experimental groups, the model was used to evaluate the main effects of plant material and treatment and the plant material × treatment interaction. Following significant effects, Tukey's honestly significant difference (HSD) test was used for pairwise comparisons among the four plant material × treatment means at *P* < 0.05. Superscript letters in Tables 3 and 4 indicate Tukey HSD groupings generated using the same ANOVA error term for the corresponding analyte. Means sharing at least one letter were not significantly different, whereas means with no common letter differed significantly at *P* < 0.05.

**Table 3.** Effect of simulated herbivory on glucose and fructose concentrations in NERICA cultivar 9 rice and EpuriPur sorghum.

Plant material	Treatment	Glucose (μg g^−1^ FW)	Fructose (μg g^−1^ FW)
NERICA 9 rice	Untreated Control	1.41 ± 0.003^c^	1.22 ± 0.001^c^
NERICA 9 rice	WOS	0.95 ± 0.001^d^	0.76 ± 0.002^d^
Epuripur sorghum	Untreated Control	2.90 ± 0.001^a^	2.50 ± 0.003^a^
Epuripur sorghum	WOS	2.30 ± 0.002^b^	1.90 ± 0.003^b^

Note: Values are means ± SE calculated from three independent biological replicates (*n* = 3 plants per plant material × treatment combination). Where replicate HPLC injections were performed, injections from each individual plant were averaged before calculation of the biological replicate mean. Within each analyte, means followed by different superscript letters differ significantly according to Tukey's HSD test at *P* < 0.05; means sharing at least one letter are not significantly different. WOS, mechanical leaf wounding followed by application of diluted *Mythimna loreyi* oral secretions.

**Table 4.** Phenolic acid concentrations in untreated and WOS-treated rice and sorghum.

Phenolic compound	NERICA 9 Untreated Control	NERICA 9 WOS	Epuripur sorghum Untreated Control	Epuripur sorghum WOS
*p*-Hydroxybenzoic acid	0.062 ± 0.000^c^	0.234 ± 0.012^b^	0.082 ± 0.020^c^	0.325 ± 0.010^a^
Vanillic acid	2.389 ± 0.002^d^	7.515 ± 0.010^b^	4.235 ± 0.005^c^	8.624 ± 0.020^a^
Caffeic acid	ND	ND	ND	ND
Syringic acid	0.295 ± 0.040^d^	1.121 ± 0.020^c^	1.295 ± 0.010^b^	2.805 ± 0.010^a^
*p*-Coumaric acid	ND^d^	0.442 ± 0.030^c^	0.842 ± 0.020^b^	1.199 ± 0.040^a^
Cinnamic acid	ND^a^	2.546 ± 0.030^b^	ND^a^	5.130 ± 0.020^c^
Chlorobenzoic acid	1.056 ± 0.040^c^	2.240 ± 0.030^b^	ND^d^	3.036 ± 0.020^a^

Note: Values are means ± SE calculated from three independent biological replicates per plant material × treatment combination. ND indicates that the analyte was not detected under the analytical conditions used and does not establish complete absence. Caffeic acid was not detected in any group and was excluded from inferential analysis. No zero concentration was assigned to ND observations.

Tukey HSD controlled the family-wise error rate for the pairwise comparisons conducted within each individual analyte. Glucose, fructose, and the individual phenolic acids were analyzed as separate response variables; therefore, no additional multiplicity adjustment was applied across the different metabolites. Accordingly, the reported *P* values represent analyte-specific inferential results rather than a study-wide error-controlled set of metabolite discoveries. The findings were therefore interpreted in the context of the targeted and relatively small set of biochemical variables examined.

Non-detected (ND) observations were treated as below-detection observations rather than as confirmed zero concentrations. Accordingly, ND values were not assigned arbitrary numerical concentrations for inferential statistical analysis. Caffeic acid, which was not detected in any experimental group, was summarized descriptively and excluded from inferential analysis.

For *p*-coumaric acid, cinnamic acid, and chlorobenzoic acid, detection patterns were reported separately from quantified concentrations. Because the number of biological replicates was small and the phenolic-acid detection limits had not been formally established, inferential comparisons involving these analytes were interpreted cautiously. No statistical significance was inferred solely from differences between detected and non-detected observations. Concentration-based comparisons were considered only where the available quantified data supported a valid analysis. The limitations associated with non-detection and the small sample size were considered when interpreting treatment effects.

Given the relatively small number of biological replicates (*n* = 3 per experimental group as reported in Table 3 and supplementary Table 1), the ANOVA and Tukey HSD results were used to characterize differences among the experimental groups under the conditions of the study rather than to support broad population-level inference (Table 5). The small sample size also limits the precision of effect estimates and the assessment of distributional assumptions. Where broader metabolomic or multi-analyte screening is undertaken, a prespecified strategy for controlling multiplicity across response variables would also be appropriate.

**Table 5.** Two-way ANOVA of biochemical responses of NERICA 9 rice and Epuripur sorghum to simulated caterpillar herbivory.

Biochemical variable	Source	df	SS	MS	F	P
Glucose	Plant material	1	6.0492	6.0492	537,706.67	<0.001
	Treatment	1	0.8427	0.8427	74,906.67	<0.001
	Plant material × Treatment	1	0.0147	0.0147	1,306.67	<0.001
	Error	8	0.00009	0.000011	–	–
Fructose	Plant material	1	4.3923	4.3923	254,626.09	<0.001
	Treatment	1	0.8427	0.8427	48,852.17	<0.001
	Plant material × Treatment	1	0.0147	0.0147	852.17	<0.001
	Error	8	0.000138	0.000017	–	–
*p*-Hydroxybenzoic acid	Plant material	1	0.009241	0.009241	19.132	0.002
	Treatment	1	0.129169	0.129169	267.430	<0.001
	Plant material × Treatment	1	0.003781	0.003781	7.828	0.023
	Error	8	0.003864	0.000483	–	–
Vanillic acid	Plant material	1	6.549019	6.549019	16,506.664	<0.001
	Treatment	1	67.901419	67.901419	171,144.093	<0.001
	Plant material × Treatment	1	0.407377	0.407377	1,026.784	<0.001
	Error	8	0.003174	0.000397	–	–
Syringic acid	Plant material	1	5.402892	5.402892	3,274.480	<0.001
	Treatment	1	4.092672	4.092672	2,480.407	<0.001
	Plant material × Treatment	1	0.350892	0.350892	212.662	<0.001
	Error	8	0.013200	0.001650	–	–
*p*-Coumaric acid	Plant material	1	1.917601	1.917601	672.842	<0.001
	Treatment	1	0.478801	0.478801	168.000	<0.001
	Plant material × Treatment	1	0.005419	0.005419	1.901	0.205
	Error	8	0.022800	0.002850	–	–
Cinnamic acid	Plant material	1	5.007792	5.007792	5,136.197	<0.001
	Treatment	1	44.190732	44.190732	45,323.828	<0.001
	Plant material × Treatment	1	5.007792	5.007792	5,136.197	<0.001
	Error	8	0.007800	0.000975	–	–
Chlorobenzoic acid	Plant material	1	0.050700	0.050700	23.310	0.001
	Treatment	1	13.356300	13.356300	6,140.828	<0.001
	Plant material × Treatment	1	2.572428	2.572428	1,182.726	<0.001
	Error	8	0.017400	0.002175	–	–

Note: Two-way ANOVA was performed with plant material (NERICA 9 rice and Epuripur sorghum) and treatment (control and WOS) as fixed factors. WOS denotes mechanical leaf wounding followed by application of diluted *Mythimna loreyi* oral secretions. Each plant material × treatment combination comprised three independent biological replicates. Error degrees of freedom = 8. Caffeic acid was not detected in any experimental group and was therefore excluded from ANOVA. ND observations for analytes in which non-detection occurred were assigned a value of zero for the statistical analysis; this substitution should be considered when interpreting the corresponding tests.

## Results

### HPLC method performance and identification of targeted metabolites

The HPLC analytical procedures enabled detection of the targeted soluble sugars and phenolic acids in leaf extracts of NERICA 9 rice and Epuripur sorghum. Glucose and fructose showed strong calibration linearity over the concentration range of 0.5–20 μg L^−1^, with coefficients of determination of R^2^ = 0.999 for both analytes. The estimated limits of detection were 0.01 μg L^−1^ for glucose and 0.03 μg L^−1^ for fructose (Table 1). These results indicate consistent calibration performance for the two soluble sugars within the investigated concentration range.

The targeted phenolic acids were identified by comparison of their chromatographic retention times with those of authentic standards. The experimentally determined retention times of reference standards were 19.403 min for *p*-hydroxybenzoic acid, 31.520 min for vanillic acid, 32.491 min for caffeic acid, 33.899 min for syringic acid, 35.147 min for *p*-coumaric acid, 43.243 min for cinnamic acid, and 46.496 min for chlorobenzoic acid (Table 1 and Figure 1). Caffeic acid was not detected in either plant material under the analytical conditions used (Table 4, Figure 2), and was therefore not included in the two-way ANOVA or Tukey HSD comparisons (Table 5).

### Changes in soluble sugars following simulated herbivory

The two-way ANOVA indicated significant effects of plant material, treatment, and their interaction on both glucose and fructose concentrations (Table 5; *P* < 0.001 for all three effects). Thus, the measured concentrations differed between the two plant materials, and the magnitude of the treatment-associated response varied between them under the statistical model used.

For glucose, NERICA 9 rice contained 1.41 ± 0.003 μg g^−1^ FW in untreated plants and 0.95 ± 0.001 μg g^−1^ FW following WOS treatment. Epuripur sorghum contained 2.90 ± 0.001 μg g^−1^ FW in untreated plants and 2.30 ± 0.002 μg g^−1^ FW following WOS treatment (Table 3 and supplementary Table 1). The four plant material × treatment combinations were assigned different Tukey HSD groupings, indicating significant differences among the means. Glucose concentration was therefore highest in untreated Epuripur sorghum and lowest in WOS-treated NERICA 9.

Fructose showed a similar pattern. In NERICA 9, the concentration decreased from 1.22 ± 0.001 to 0.76 ± 0.002 μg g^−1^ FW following WOS treatment, whereas in Epuripur sorghum it decreased from 2.50 ± 0.003 to 1.90 ± 0.003 μg g^−1^ FW (Table 3 and supplementary Table 1). The different superscript groupings indicate significant differences among the four plant material × treatment combinations.

The significant plant material × treatment interactions for glucose and fructose indicate that the magnitude of the WOS-associated response differed between NERICA 9 and Epuripur sorghum. Although both materials showed lower concentrations following WOS treatment, glucose and fructose concentrations remained higher in Epuripur sorghum under both treatment conditions.

### Phenolic-acid responses in NERICA 9 rice

WOS treatment was associated with changes in several targeted phenolic acids in NERICA 9 rice (Table 4). *p*-Hydroxybenzoic acid increased from 0.062 ± 0.000 μg g^−1^ FW in untreated plants to 0.234 ± 0.012 μg g^−1^ FW following WOS treatment. Vanillic acid increased from 2.389 ± 0.002 to 7.515 ± 0.010 μg g^−1^ FW, while syringic acid increased from 0.295 ± 0.040 to 1.121 ± 0.020 μg g^−1^ FW.

*p*-Coumaric acid was not detected in untreated NERICA 9 but was detected at 0.442 ± 0.030 μg g^−1^ FW following WOS treatment. Cinnamic acid was similarly not detected in untreated NERICA 9 but was detected at 2.546 ± 0.030 μg g^−1^ FW following WOS treatment. Chlorobenzoic acid was detected under both conditions and increased from 1.056 ± 0.040 to 2.240 ± 0.030 μg g^−1^ FW. Caffeic acid was not detected in either treatment.

The two-way ANOVA showed significant main effects of plant material and WOS treatment for *p*-hydroxybenzoic acid, vanillic acid, syringic acid, *p*-coumaric acid, cinnamic acid, and chlorobenzoic acid (Table 5). Significant plant material × treatment interactions were observed for *p*-hydroxybenzoic acid (*P* = 0.023), vanillic acid (*P* < 0.001), syringic acid (*P* < 0.001), cinnamic acid (*P* < 0.001), and chlorobenzoic acid (*P* < 0.001). In contrast, the interaction for *p*-coumaric acid was not statistically significant (*P* = 0.205). Thus, although *p*-coumaric acid showed significant main effects of plant material and treatment, the evidence did not support a statistically significant difference in the treatment response between the two plant materials.

### Phenolic-acid responses in Epuripur Sorghum

Epuripur sorghum also showed treatment-associated changes in several targeted phenolic acids (Table 4). *p*-Hydroxybenzoic acid increased from 0.082 ± 0.020 to 0.325 ± 0.010 μg g^−1^ FW following WOS treatment. Vanillic acid increased from 4.235 ± 0.005 to 8.624 ± 0.020 μg g^−1^ FW, while syringic acid increased from 1.295 ± 0.010 to 2.805 ± 0.010 μg g^−1^ FW.

The concentration of *p*-coumaric acid increased from 0.842 ± 0.020 to 1.199 ± 0.040 μg g^−1^ FW following WOS treatment, with the two Epuripur sorghum means assigned different Tukey HSD groupings (Table 4). In NERICA 9, *p*-coumaric acid was not detected in untreated plants but was detected at 0.442 ± 0.030 μg g^−1^ FW following WOS treatment. The plant material × treatment interaction for *p*-coumaric acid was not significant (*P* = 0.205), indicating that the statistical evidence did not support a difference in treatment response between the two plant materials despite the significant main effects.

Cinnamic acid was not detected in untreated Epuripur sorghum but was detected at 5.130 ± 0.020 μg g^−1^ FW following WOS treatment. In Epuripur sorghum, chlorobenzoic acid was not detected in untreated plants but was detected at 3.036 ± 0.020 μg g^−1^ FW following WOS treatment. The four plant material × treatment combinations were assigned distinct Tukey HSD groupings (Table 4), consistent with the significant plant material × treatment interaction (*P* < 0.001). Caffeic acid was not detected in either treatment.

The two-way ANOVA indicated significant effects of plant material and treatment for *p*-hydroxybenzoic acid, vanillic acid, syringic acid, *p*-coumaric acid, cinnamic acid, and chlorobenzoic acid (Table 5). Significant plant material × treatment interactions were detected for *p*-hydroxybenzoic acid, vanillic acid, syringic acid, cinnamic acid, and chlorobenzoic acid, whereas the interaction for *p*-coumaric acid was not significant (*P* = 0.205).

### Comparative biochemical responses of NERICA 9 and Epuripur Sorghum

The two plant materials exhibited a broadly similar direction of biochemical response to WOS treatment, characterized by decreases in glucose and fructose and increases or treatment-associated detection of several phenolic acids. However, the magnitude and absolute concentrations of these responses differed between the two materials.

Epuripur sorghum contained higher glucose and fructose concentrations than NERICA 9 under both control and WOS conditions. It also contained higher concentrations of vanillic and syringic acids under both experimental conditions. For *p*-hydroxybenzoic acid, vanillic acid, syringic acid, cinnamic acid, and chlorobenzoic acid, significant plant material × treatment interactions indicate that the response to WOS depended on the plant material examined.

For *p*-coumaric acid, significant main effects of plant material and treatment occurred in the absence of a significant interaction (*P* = 0.205). Thus, the overall differences in *p*-coumaric acid were statistically associated with plant material and treatment, but there was insufficient evidence that the magnitude of the treatment effect differed significantly between NERICA 9 and Epuripur sorghum.

The calibration results demonstrate strong linearity for glucose and fructose over the investigated standard range and provide estimated detection limits for these two analytes. These analytical-performance observations apply only to the soluble-sugar measurements and should not be interpreted as complete validation of the targeted phenolic-acid measurements.

Overall, the biochemical profiles demonstrated substantial differences between the two specific plant materials examined. These findings should therefore be interpreted as genotype/material-specific responses rather than as evidence of species-wide differences between sorghum and rice. The two-way ANOVA demonstrated significant effects of plant material and treatment for the principal measured biochemical variables and significant plant material × treatment interactions for glucose, fructose, *p*-hydroxybenzoic acid, vanillic acid, syringic acid, cinnamic acid, and chlorobenzoic acid. The interaction for *p*-coumaric acid was not significant (*P* = 0.205).

## Discussion

### Simulated herbivory was associated with coordinated changes in primary and phenolic metabolism

The present study demonstrated that combined mechanical wounding and *Mythimna loreyi* oral-secretory application was associated with significant changes in soluble sugars and several targeted phenolic acids in NERICA 9 rice and Epuripur sorghum. The two-way ANOVA showed significant effects of plant material and treatment for several quantified biochemical variables, with significant plant material × treatment interactions for glucose, fructose, *p*-hydroxybenzoic acid, vanillic acid, syringic acid, cinnamic acid, and chlorobenzoic acid (Table 5).

These findings are consistent with the established capacity of herbivore-associated damage to alter both primary and specialized metabolism. Mechanical injury can activate intracellular calcium signaling, reactive oxygen species, mitogen-activated protein kinase cascades, and phytohormonal pathways involving jasmonates, ethylene, and salicylic acid.^8‐10^ Herbivore oral secretions can further modify wound-induced responses through elicitor-associated signaling.^11^ ^,^ ^12^

Nevertheless, the treatment used in the present study combined mechanical injury and oral secretions. Consequently, the observed biochemical changes represent responses to the combined WOS treatment and cannot be attributed specifically to *M. loreyi* oral-secretory components. Wounding-only and oral-secretion-only treatments would be required to distinguish their independent and interactive effects.

### WOS treatment was associated with reduced soluble sugar concentrations

Both glucose and fructose decreased following WOS treatment in NERICA 9 and Epuripur sorghum. The ANOVA showed significant treatment effects for glucose and fructose (*P* < 0.001 for both), together with significant plant material × treatment interactions (*P* < 0.001; Table 5).

Changes in soluble sugar pools following herbivore-associated stress are biologically plausible because carbohydrates function both as metabolic substrates and as signaling molecules. Herbivore attack can alter respiratory demand, biosynthetic activity, source–sink relationships, transport, and other components of primary metabolism.^16^ ^,^ ^24^

The reductions in glucose and fructose could therefore reflect changes in carbohydrate utilization or partitioning following tissue damage. However, the present measurements represent metabolite concentrations at a single sampling point and do not establish the mechanism responsible for the observed decreases.

In particular, the lower sugar concentrations should not be interpreted as direct evidence that carbon was reallocated from growth to defense. Demonstrating carbon allocation would require measurements such as photosynthetic carbon assimilation, respiration, biomass partitioning, carbon flux, or isotopic tracing. Similarly, because plant growth was not measured, the results do not demonstrate a growth–defense trade-off.^25^

### Material-specific differences in soluble sugars

Epuripur sorghum maintained higher concentrations of glucose and fructose than NERICA 9 under both control and WOS conditions. The significant plant material effects for both sugars (*P* < 0.001) support biochemical differences between the two experimental materials (Table 5).

The C₄ photosynthetic pathway of sorghum and C₃ pathway of rice provide relevant physiological context for considering differences in primary metabolism.^26^ ^,^ ^27^ C₃ and C₄ plants differ in photosynthetic carbon fixation, photorespiration, and associated metabolic organization. Nevertheless, photosynthetic rates and carbon assimilation were not measured in the present experiment. Therefore, the higher sugar concentrations in Epuripur sorghum cannot be attributed specifically to C₄ photosynthesis.

Other factors, including genetic background, developmental stage, tissue composition, growth conditions, and constitutive differences in carbohydrate metabolism, could also contribute to the observed differences. The most direct interpretation is therefore that Epuripur sorghum and NERICA 9 differed in their measured soluble-sugar pools under the conditions of this experiment.

### WOS treatment was associated with changes in phenolic-acid profiles

Several phenolic acids showed treatment-associated increases or became detectable following WOS treatment. In both materials, *p*-hydroxybenzoic acid, vanillic acid, and syringic acid increased following treatment. Cinnamic acid was detected following WOS treatment but not in untreated plants in both materials, while *p*-coumaric acid was detected after treatment in NERICA 9 and increased numerically in Epuripur sorghum.

Phenolic compounds have diverse functions in plant responses to biotic stress, including antioxidant activity, modification of cell-wall-associated properties, and interactions with herbivores and microorganisms ^18‐23^. Several of the compounds measured in this study are also associated with phenylpropanoid metabolism.

The treatment-associated changes are therefore consistent with modification of phenolic metabolism following simulated herbivory. However, metabolite accumulation alone does not demonstrate increased pathway flux. Simulated herbivory was associated with higher reported concentrations of several phenolic acids in rice and sorghum. However, interpretation of *p*-coumaric acid, cinnamic acid, and chlorobenzoic acid requires particular caution because these compounds were not detected in some experimental groups. Non-detection indicates that concentrations were below the analytical detection capability and does not necessarily indicate complete absence. Consequently, differences between ND and quantified observations cannot be interpreted as precise quantitative changes without considering the analytical detection threshold. Caffeic acid was not detected in any group. Because the study included only three independent biological replicates per treatment combination, the results for analytes affected by non-detection are presented primarily as descriptive evidence. These limitations do not negate the observed concentration patterns in detected samples but restrict the certainty with which treatment effects can be inferred. The present biochemical observations complement our recent findings on sorghum volatile-mediated responses to simulated herbivory. Osinde et al.^17^ demonstrated that mechanically wounded sorghum treated with *Chilo partellus* oral secretions emitted candidate herbivore-induced volatiles, including DMNT and (Z)-3-hexenyl acetate, and that these compounds elicited behavioral responses in *Cotesia flavipes*. Together, these findings indicate that herbivore-associated stimulation can be accompanied by changes in both internal metabolite profiles and externally emitted chemical signals. However, the two studies examined different response classes and experimental systems; the present study measured soluble sugars and targeted phenolic acids following *Mythimna loreyi* oral-secretory treatment, whereas Osinde et al.^17^ examined volatile emissions and parasitoid behavioral responses following *C. partellus* oral-secretory treatment. The studies therefore provide complementary rather than directly equivalent evidence.

### Phenolic Responses in NERICA 9 rice

NERICA 9 showed substantial increases in several phenolic acids following WOS treatment. Vanillic acid increased from 2.389 to 7.515 μg g^−1^ FW, while syringic acid increased from 0.295 to 1.121 μg g^−1^ FW. *p*-Hydroxybenzoic acid increased from 0.062 to 0.234 μg g^−1^ FW.

The detection of *p*-coumaric acid and cinnamic acid following WOS treatment, when these compounds were not detected in untreated NERICA 9, indicates a treatment-associated change in the detectable phenolic-acid profile. Chlorobenzoic acid was detected under both conditions and showed a numerical increase from 1.056 to 2.240 μg g^−1^ FW.

The significant treatment effects for these phenolic variables are compatible with an induced biochemical response to the combined wounding and oral-secretory treatment. Rice possesses well-characterized inducible defense responses involving herbivore-associated signaling and metabolic changes.^13^ ^,^ ^35^

However, the present study cannot determine whether the observed changes resulted from increased biosynthesis, reduced degradation, altered transport, or other metabolic processes. The endpoint concentrations should therefore be interpreted as biochemical responses rather than direct measurements of pathway activation.

### Phenolic responses in Epuripur Sorghum

Epuripur sorghum also exhibited substantial changes in its targeted phenolic-acid profile. Vanillic acid increased from 4.235 to 8.624 μg g^−1^ FW, syringic acid from 1.295 to 2.805 μg g^−1^ FW, and *p*-hydroxybenzoic acid from 0.082 to 0.325 μg g^−1^ FW.

*p*-Coumaric acid increased from 0.842 to 1.199 μg g^−1^ FW in Epuripur sorghum following WOS treatment, and the two means differed according to the Tukey HSD grouping. In NERICA 9, *p*-coumaric acid was not detected in untreated plants but was detected following WOS treatment. Nevertheless, the plant material × treatment interaction was not significant (*P* = 0.205). Thus, the evidence supports effects of plant material and treatment on *p*-coumaric acid concentrations but does not establish that the magnitude of the treatment response differed significantly between the two materials.

Cinnamic acid was detected at 5.130 μg g^−1^ FW following WOS treatment but was not detected in untreated plants. Chlorobenzoic acid similarly became detectable following treatment, reaching 3.036 μg g^−1^ FW.

The significant treatment and plant material effects observed for the phenolic compounds indicate that WOS treatment was associated with changes in the phenolic profile of Epuripur. Sorghum is known to contain diverse phenolic and other specialized metabolites that can vary with genotype and environmental conditions.^22^ ^,^ ^28^

The present observations complement earlier work showing that sorghum and rice can differ in their volatile responses to artificial herbivory.^29^ More recently, Osinde et al.^17^ demonstrated that selected sorghum herbivore-induced volatiles, particularly DMNT and (Z)-3-hexenyl acetate, were associated with behavioral attraction of *Cotesia flavipes*. The current findings extend this research direction from volatile-mediated signaling to changes in soluble sugars and targeted phenolic acids, providing a complementary biochemical perspective on the response of sorghum to herbivore-associated stimulation.

### Plant material × treatment interactions indicate material-dependent responses

The significant interaction terms in Table 5 provide important comparative information. Significant plant material × treatment interactions were observed for glucose, fructose, *p*-hydroxybenzoic acid, vanillic acid, syringic acid, cinnamic acid, and chlorobenzoic acid. These interactions indicate that the magnitude of the WOS-associated response differed between the two plant materials.

For glucose and fructose, both materials showed reductions following WOS treatment, but Epuripur sorghum retained higher absolute concentrations. For vanillic acid and syringic acid, both materials showed increases, but their starting concentrations and treatment-associated concentrations differed considerably.

For *p*-coumaric acid, the interaction was not significant (*P* = 0.205), despite significant main effects of plant material and treatment. This distinction is important because it indicates that the overall differences in *p*-coumaric acid should not be interpreted as evidence that the treatment response differed significantly between the two materials.

The significant interactions observed for the other compounds support a material-dependent interpretation rather than a uniform biochemical response to WOS across the two cereals.

### Relationship between soluble sugars and phenolic metabolism

The simultaneous decrease in soluble sugars and increase in several phenolic acids suggests that WOS treatment was associated with changes in both primary and specialized metabolism. Such coordinated changes are compatible with metabolic adjustment following tissue damage and herbivore-associated stimulation.

However, the data do not demonstrate that glucose or fructose was directly converted into phenolic compounds. No metabolic-flux analysis, isotopic tracing, enzyme assay, transcriptomic analysis, or direct carbon-allocation measurement was performed. Furthermore, no correlation analysis between sugar and phenolic concentrations was conducted.

Consequently, the results demonstrate parallel changes in two metabolite classes rather than a demonstrated biochemical transfer of carbon from soluble sugars into phenolic compounds.

### Treatment-responsive phenolic acids as candidates for further investigation

The treatment-associated changes in *p*-hydroxybenzoic acid, vanillic acid, syringic acid, *p*-coumaric acid, cinnamic acid, and chlorobenzoic acid identify metabolites that warrant further investigation.

These compounds should, however, be regarded as candidate treatment-responsive metabolites rather than validated biochemical markers of insect resistance. A resistance-associated marker would require consistent association between metabolite abundance and a defined resistance phenotype, preferably supported by direct measurements of insect feeding, larval growth, survival, development, or reproduction.

A metabolite may increase following herbivore-associated stimulation without necessarily reducing herbivore performance. Therefore, the present biochemical measurements cannot establish whether any of the detected compounds contribute functionally to resistance.

### Comparative interpretation of Epuripur Sorghum and NERICA 9 rice

The comparison demonstrated clear biochemical differences between Epuripur sorghum and NERICA 9. Epuripur sorghum generally contained higher concentrations of glucose, fructose, vanillic acid, and syringic acid under both control and WOS conditions. Several treatment-associated differences were also reflected in significant plant material × treatment interactions.

These findings indicate that the two experimental materials differed in their constitutive metabolite pools and/or their biochemical responses to WOS treatment. They do not establish that sorghum as a species possesses greater defensive capacity than rice.

Only one sorghum cultivar and one rice material were evaluated, and the experiment was conducted under a single greenhouse environment and at a single post-treatment sampling time. Genetic variation within both crops could therefore be substantial. Additional genotypes and environments would be required to determine whether the observed patterns are consistently associated with crop type or instead reflect characteristics of the particular materials tested.

### Interpretation of the simulated-herbivory treatment

The WOS treatment combined mechanical leaf damage with application of diluted *M. loreyi* oral secretions. This approach provides a controlled means of examining biochemical responses associated with caterpillar-like stimulation without requiring continuous larval feeding.

Nevertheless, the design included only untreated and WOS treatments. It therefore cannot distinguish the effects of mechanical injury from those of oral secretions or determine whether the two components interact.

This distinction is particularly important because *M. loreyi* was used as the source of oral secretions, whereas no actual stem-borer feeding occurred. The experiment therefore provides evidence about biochemical responses to simulated caterpillar-associated stimulation rather than direct resistance to stem borers.

### Implications for crop improvement and future research

The metabolites identified in this study provide candidates for subsequent biochemical screening of cereal germplasm. Vanillic acid, syringic acid, *p*-coumaric acid, cinnamic acid, *p*-hydroxybenzoic acid, and chlorobenzoic acid could be evaluated across larger panels of sorghum and rice genotypes.

Integrating biochemical profiling with transcriptomics, metabolomics, physiological measurements, genetic analysis, and insect-performance assays would provide stronger evidence concerning the biological significance of these metabolites.^16^ ^,^ ^36^

For sorghum, the substantial genetic diversity available within the crop provides an opportunity to investigate whether variation in biochemical responses is genetically controlled and associated with resistance-related phenotypes.^37^ ^,^ ^38^ Comparable analyzes across diverse rice materials would allow determination of whether the patterns observed here are reproducible.

### Analytical validation and scope limitation

An additional limitation concerns the analytical scope and validation of the HPLC measurements. The phenolic analysis was targeted to selected phenolic acids rather than the complete phenolic metabolome; therefore, compounds not detected under the chromatographic conditions cannot be assumed to be completely absent from the plant tissue. The available analytical-performance assessment established strong calibration linearity and estimated detection limits for glucose and fructose, but a complete validation according to ICH Q2(R2) was not performed for all analytes.^34^ In particular, formal precision, accuracy/recovery, LOQ, and related validation characteristics were not established for the targeted phenolic acids. Accordingly, the phenolic-acid results should be interpreted as targeted quantitative measurements obtained under the specified chromatographic conditions rather than as measurements generated by a fully validated multi-analyte method. Future work should include formal validation of the phenolic-acid method, including analyte-specific LOD and LOQ, intra- and inter-day precision, accuracy/recovery, calibration performance, and appropriate specificity assessments.^34^

### Limitations and future directions

Several limitations should be considered. First, the experiment included only untreated and WOS treatments; consequently, the individual effects of mechanical injury and oral secretions could not be separated. Second, actual herbivore feeding and insect-performance measurements were not included. Third, only Epuripur sorghum and NERICA 9 rice were evaluated, limiting extrapolation to other genotypes.

Fourth, measurements were made at a single 24-h sampling point, preventing assessment of temporal changes in metabolite accumulation. Fifth, soluble-sugar concentrations were interpreted as changes in metabolite pools rather than direct evidence of carbon allocation because photosynthesis, respiration, biomass partitioning, and carbon flux were not measured.

The statistical interpretation also requires attention to the significant interaction terms. In particular, main effects should not be interpreted independently where a significant plant material × treatment interaction is present. The Tukey groupings in Tables 3 and 4 and 4 should be retained only if they correspond exactly to the post-hoc comparisons generated from the same ANOVA model.

Future experiments should therefore include at least four treatments: untreated control, mechanical wounding alone, oral secretions alone, and combined mechanical wounding plus oral secretions. Incorporation of actual herbivore feeding, multiple genotypes, multiple sampling times, greater biological replication, photosynthetic measurements, carbon-flux analyzes, and insect-performance assays would further clarify the mechanisms underlying the observed responses.

### Overall interpretation

Taken together, the results demonstrate that combined mechanical wounding and *M. loreyi* oral-secretory application was associated with substantial biochemical changes in the tested NERICA 9 rice and Epuripur sorghum materials. Glucose and fructose concentrations were lower following WOS treatment in both materials, whereas several targeted phenolic acids increased or became detectable following treatment.

The two-way ANOVA demonstrated significant effects of plant material and treatment for the principal measured biochemical variables and significant plant material × treatment interactions for glucose, fructose, *p*-hydroxybenzoic acid, vanillic acid, syringic acid, cinnamic acid, and chlorobenzoic acid. The interaction for *p*-coumaric acid was not significant.

These findings indicate that the biochemical response to WOS varied among metabolites and, for several compounds, depended on the plant material examined. Epuripur sorghum generally maintained higher concentrations of several measured metabolites than NERICA 9, but this observation is specific to the two materials tested and should not be generalized to the species as a whole.

The decrease in soluble sugars is consistent with altered carbohydrate metabolism but does not demonstrate carbon reallocation or a growth–defense trade-off. Likewise, increases in phenolic acids indicate changes in the measured phenolic profile but do not establish direct effects on insect performance or resistance.

The findings therefore provide a biochemical characterization of responses to simulated caterpillar herbivory in two cereal materials and identify several phenolic acids for subsequent functional investigation. Experiments incorporating wounding-only and oral-secretion-only controls, actual herbivore feeding, diverse genotypes, time-course sampling, physiological measurements, and insect-performance assays will be necessary to determine the mechanisms and functional significance of these biochemical responses.

## Conclusion

This study shows that simulated caterpillar herbivory, consisting of mechanical leaf wounding combined with *Mythimna loreyi* oral secretions, was associated with changes in soluble sugar and phenolic-acid profiles in the tested Epuripur sorghum and NERICA 9 rice materials. Glucose and fructose concentrations were significantly lower following WOS treatment in both materials, whereas several phenolic acids showed numerical increases. The magnitude and statistical support of these responses varied among metabolites and crop materials.

The two plant materials differed in their baseline and treatment-associated metabolite concentrations, with Epuripur sorghum generally showing higher concentrations of several measured metabolites. These differences should be interpreted as biochemical differences between the specific materials examined rather than evidence of greater species-wide defensive capacity in sorghum.

The decline in soluble sugars is consistent with altered carbohydrate metabolism but does not demonstrate carbon reallocation toward defense or a growth–defense trade-off. Similarly, increased phenolic concentrations indicate metabolic responses associated with simulated herbivory but do not establish direct resistance to stem borers or other herbivores.

A major limitation is that the experimental design included only untreated and WOS treatments; therefore, mechanical-wounding and oral-secretory effects could not be separated. Likewise, the experiment did not directly evaluate stem-borer resistance because *M. loreyi* oral secretions and artificial leaf wounding were used instead of actual stem-borer feeding. The findings are therefore best interpreted as biochemical responses to artificial caterpillar-associated stimulation.

The treatment-associated changes in *p*-hydroxybenzoic, vanillic, syringic, *p*-coumaric, cinnamic, and chlorobenzoic acids identify candidate metabolites for further investigation. Because the phenolic analysis was targeted and was not fully validated according to ICH Q2(R2), non-detection should be interpreted within the analytical conditions used rather than as evidence of complete absence from the tissue.^34^

Overall, the study provides evidence that the tested sorghum and rice materials undergo distinct biochemical changes following artificial caterpillar-associated stimulation and provides a basis for integrating biochemical profiling with physiological, molecular, and insect-performance approaches to investigate cereal defense.

Future studies incorporating wounding-only and oral-secretion-only treatments, actual herbivore feeding, larger numbers of biological replicates, measurements of photosynthesis and carbon allocation, and insect-performance assays would help resolve the mechanisms underlying the observed biochemical responses and establish their functional significance in herbivore resistance.

## Supplementary Material

Supplementary Material Supplementary_Table__OF_KPSB-S-2026-0306.R2__2_.docx
