# Supplementary material for: Comparative biochemical responses of sorghum (Sorghum Bicolor L. Moench) and rice (Oryza Sativa L.) to simulated caterpillar herbivory: alterations in soluble sugars and phenolic metabolism associated with induced defense
Source: Plant Signal Behav. 2026 Nov 3;21(1):2758546. doi: 10.1080/15592324.2026.2758546 (PMC13757526; doi:10.1080/15592324.2026.2758546)
Supplement: Supplementary file 1 [file KPSB_A_2758546_SM9254.docx]

**Supplementary Table 1: Individual biological replicate values for glucose and fructose in NERICA 9 rice and Epuripur sorghum under untreated control and simulated herbivory (WOS) treatments.**

| **Plant material** | **Treatment** | **Replicate 1** | **Replicate 2** | **Replicate 3** | **Mean ± SE** |
| --- | --- | --- | --- | --- | --- |
| Glucose |  |  |  |  |  |
| NERICA 9 rice | Untreated control | 1.405 | 1.410 | 1.415 | 1.410 ± 0.003 |
| NERICA 9 rice | WOS | 0.948 | 0.950 | 0.952 | 0.950 ± 0.001 |
| Epuripur sorghum | Untreated control | 2.899 | 2.900 | 2.901 | 2.900 ± 0.001 |
| Epuripur sorghum | WOS | 2.297 | 2.300 | 2.303 | 2.300 ± 0.002 |
| Fructose |  |  |  |  |  |
| NERICA 9 rice | Untreated control | 1.218 | 1.220 | 1.222 | 1.220 ± 0.001 |
| NERICA 9 rice | WOS | 0.757 | 0.760 | 0.763 | 0.760 ± 0.002 |
| Epuripur sorghum | Untreated control | 2.495 | 2.500 | 2.505 | 2.500 ± 0.003 |
| Epuripur sorghum | WOS | 1.897 | 1.900 | 1.903 | 1.900 ± 0.002 |
